# Proteins from Microalgae: Nutritional, Functional and Bioactive Properties

**DOI:** 10.3390/foods14060921

**Published:** 2025-03-08

**Authors:** Juan Pablo García-Encinas, Saul Ruiz-Cruz, Jousé Juárez, José de Jesús Ornelas-Paz, Carmen Lizette Del Toro-Sánchez, Enrique Márquez-Ríos

**Affiliations:** 1 Departamento de Investigación y Posgrado en Alimentos, Universidad de Sonora, Boulevard Luis Encinas y Rosales, Hermosillo 83000, Sonora, Mexico; a218211462@unison.mx (J.P.G.-E.); saul.ruizcruz@unison.mx (S.R.-C.); carmen.deltoro@unison.mx (C.L.D.T.-S.); 2 Departamento de Física, Universidad de Sonora, Hermosillo 83000, Sonora, Mexico; josue.juarez@unison.mx; 3 Coordinación de Fisiología y Tecnología de Alimentos de la Zona Templada, Centro de Investigación en Alimentación y Desarrollo, Av. Río Conchos S/N, Parque Industrial, Cuauhtémoc 31570, Chihuahua, Mexico; jornelas@ciad.mx

**Keywords:** microalgae, proteins, extraction, nutritional properties, digestibility, functional properties

## Abstract

Microalgae have emerged as a sustainable and efficient source of protein, offering a promising alternative to conventional animal and plant-based proteins. Species such as *Arthrospira platensis* and *Chlorella vulgaris* contain protein levels ranging from 50% to 70% of their dry weight, along with a well-balanced amino acid profile rich in essential amino acids such as lysine and leucine. Their cultivation avoids competition for arable land, aligning with global sustainability goals. However, the efficient extraction of proteins is challenged by their rigid cell walls, necessitating the development of optimized methods such as bead milling, ultrasonication, enzymatic treatments, and pulsed electric fields. These techniques preserve functionality while achieving yields of up to 96%. Nutritional analyses reveal species-dependent digestibility, ranging from 70 to 90%, with *Spirulina platensis* achieving the highest rates due to low cellulose content. Functionally, microalgal proteins exhibit emulsifying, water-holding, and gel-forming properties, enabling applications in baking, dairy, and meat analogs. Bioactive peptides derived from these proteins exhibit antioxidant, antimicrobial (inhibiting *E. coli* and *S. aureus*), anti-inflammatory (reducing TNF-α and IL-6), and antiviral activities (e.g., Dengue virus inhibition). Despite their potential, commercialization faces challenges, including regulatory heterogeneity, high production costs, and consumer acceptance barriers linked to eating habits or sensory attributes. Current market products like *Spirulina*-enriched snacks and *Chlorella* tablets highlight progress, but food safety standards and scalable cost-effective extraction technologies remain critical for broader adoption. This review underscores microalgae’s dual role as a nutritional powerhouse and a source of multifunctional bioactives, positioning them at the forefront of sustainable food and pharmaceutical innovation.

## 1. Introduction

Microalgae are unicellular organisms with the most efficient photosynthetic systems in nature, achieving a photosynthetic efficiency of 10–20%, while terrestrial plants typically reach only 1–2% [1]. Their cultivation is relatively simple, as these organisms do not compete for arable land. Unlike terrestrial species, microalgae only require nutrients, can grow in non-potable water, and can be cultivated in either closed or open systems. Furthermore, contamination by bacteria or fungi can be easily avoided by cultivating microalgae in environments with elevated pH values or salinity [2,3]. These characteristics, along with their potential to yield biomolecules such as proteins, lipids, and carbohydrates, position microalgae as promising alternatives to animal-derived proteins.

Proteins are essential components of the human diet and are primarily sourced from animals. However, global population growth has led to protein shortages in certain regions or countries. As such, the need to meet the increasing demand for protein-rich foods, especially in areas with limited agricultural resources, drives the search for sustainable alternative sources to complement or replace animal-based proteins. In this context, microalgae have emerged as a promising protein source, offering a sustainable alternative to traditional animal and plant-based proteins. These microorganisms not only contain high levels of protein, but also possess unique nutritional, functional, and bioactive properties that can be applied in food, feed, and nutraceuticals. The diversity of microalgal species, estimated at around 200,000 species, presents a tremendous opportunity to discover new proteins with distinct nutritional profiles and health benefits. An example of this is the cyanobacterium *Arthrospira platensis*, which contains protein levels ranging from 50% to 70% of its dry weight, comparable to the protein content commonly found in meat. Its content also surpasses that of soy protein, which contains about 40% protein [4,5].

The choice of protein extraction method for each type of microalga is crucial, as they possess thick cell walls, necessitating the analysis of the most suitable techniques. Microalgal biomass is composed of several fractions, with the protein fraction being predominant, though it also contains other compounds, many of which are biologically active. These include carotenoids (e.g., astaxanthin and lutein), known for their antioxidant and anti-inflammatory properties; polyunsaturated fatty acids (PUFAs) like EPA and DHA, which contribute to cardiovascular and neurological health; phenolic compounds with antimicrobial and anticancer activities; and polysaccharides such as β-glucans, recognized for their immunomodulatory effects. Additionally, phycobiliproteins and chlorophyll derivatives exhibit applications, e.g., as natural pigments and photosensitizers. Such multifaceted bioactive profiles position microalgae as strategic resources, not only for biofuels and protein production, but also for nutraceuticals, pharmaceuticals, and cosmeceuticals [6]. This makes microalgae a focal point of interest for applications in biotechnology, biofuels, and cosmetics industries [7,8,9]. Additionally, peptides derived from microalgal proteins have been shown to possess bioactive properties, such as antioxidant, anti-inflammatory, antitumor, antimicrobial, antihypertensive, and immunostimulatory activities. This indicates that microalgae are not only nutritionally valuable but also have a broad range of potential applications, making them highly promising [10,11,12].

This review article will explore the main methods of protein extraction from microalgae. In addition, it will explore their nutritional properties, such as amino acid profiles; functional properties, including emulsification capacity; and bioactive properties, such as antioxidant, antimicrobial, and anti-inflammatory activities.

## 2. Microalgae Proteins

The protein content of various microalgae has been extensively studied, with several research papers published on the subject. For example, a review reported that the crude protein content in microalgal biomass ranged from 6% to 63%, and it was noted that most microalgal species contain more than 40% protein on a dry weight basis [13]. Additionally, Brown [14] conducted an analysis of protein content in different microalgae, finding that dry weight protein content varied from 12% in *Chaetoceros gracilis* to 35% in *Nannochloropsis oculata*. In another study by Becker [15] a description of the main components of different microalgae was provided, revealing a wide range of protein content (6–71% dry weight), higher than 50% in most species. Similarly, Acquah et al. [16] reported protein contents ranging from 6% to 58% across 17 different microalgal species.

All these findings demonstrate that most microalgal species contain high levels of protein, making some of them suitable for use as food proteins or dietary supplements. Examples of microalgae used for these purposes include *Chlorella* sp., *Scenedesmus obliquus*, *Arthrospira* sp., and *Spirulina* sp. [17,18,19,20,21].

Currently, microalgae-based products are already available in the market, reflecting the growing interest in this alternative source of nutrients. They are easily accessible on various e-commerce platforms, where searching for keywords like “microalgae” yields a wide range of products developed from these biomolecules, many of which are designed to benefit human health. Notable examples include “Spiruluka”, a fruit and seed bar enriched with Spirulina (*Arthrospira platensis*) and designed to provide consumers a nutrient-dense snack high in vitamins, minerals, and plant-based protein; *Chlorella* tablets, compact dietary supplements offering a concentrated source of vegan protein, essential amino acids, and detoxifying chlorophyll; and “Blue Majik” sachets, featuring a powdered formulation containing *Arthrospira platensis* extract (rich in phycocyanin, a bioactive blue pigment). These single-serving sachets are intended for mixing into beverages, enhancing their nutritional profile with antioxidants, anti-inflammatory compounds, and plant-based protein. These products exemplify the growing integration of microalgae into functional foods and supplements, aligning with global trends toward sustainable, plant-forward nutrition. The commercialization of microalgae-derived products has advanced considerably in recent years, driven by the growing interest in sustainable and nutrient-rich alternatives. However, their adoption remains uneven across different sectors, limiting their full potential. Despite the success of existing products, their incorporation into the general population’s diet continues to be a challenge due to consumer perception, as they are often considered specialized and inaccessible [2,4]. This trend not only underscores the acceptance of microalgae as functional ingredients but also highlights their potential to drive new strategies in the field of nutrition, catering to consumers seeking sustainable, healthy options aligned with environmental care.

## 3. Protein Processing Methods

One of the most significant challenges in processing microalgal proteins is their efficient extraction. This is due to the complex structure of their cell walls. Therefore, the technologies developed to break down the cell wall play a crucial role in protein extraction. Additionally, the extraction or recovery step is key to ensuring the quality, nutritional properties, and bioactivity of the proteins, as well as facilitating their application in food, nutritional supplements, and industrial products. Processing methods can range from biomass harvesting and drying to more specific techniques for recovery and purification. The choice of the appropriate processing method largely depends on the type of microalga, the characteristics of the protein, and its intended application, with the goal of minimizing the loss of biological activity during the process. Recent advancements in bioactive compound extraction have focused on developing mild methods that minimize damage while maintaining high yields. Among these strategies, enzyme-assisted extraction using proteases or carbohydrases has proven effective in breaking down rigid cell walls without compromising protein functionality. Sari et al. [22] reported a 69% protein yield from *Chlorella fusca* using tailored enzymatic treatments. Similarly, ultrasound-assisted extraction has gained attention for its ability to efficiently disrupt cell walls through cavitation forces. In this regard, Sankaran et al. [23] reported a protein extraction efficiency of 93.33% from *Chlorella vulgaris*, with minimal degradation of phycobiliproteins, which are particularly heat-sensitive. Another emerging approach combines mechanical pretreatments with enzymatic hydrolysis to enhance yields while preserving bioactivity. Mendes Costa et al. [24] demonstrated that combining lyophilization, bead milling, and trypsin treatment synergistically enhanced the release of bioactive peptides from *Chlorella vulgaris*, achieving a threefold increase in peptide concentration compared to standalone methods.

## 4. Protein Extraction Techniques

### 4.1. Mechanical Extraction

**Figure 1:**
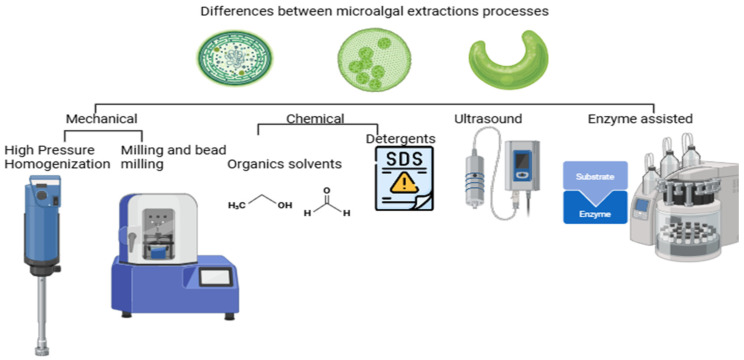
Illustrative representation of microalgal extraction processes: mechanical, chemical, and ultrasound- and enzyme-assisted methods.

Among the most common techniques are high-pressure homogenization and milling (Figure 1), which are effective at breaking down cell walls. However, these methods require large amounts of energy and, in some cases, may damage the proteins due to the shear forces involved. Mechanical cell disruption methods include cutting-force and energy transfer techniques, with bead milling being one example of a cutting-force method. In bead milling, particles are ground and dispersed to micro or nano sizes using milling and dispersion equipment. In this technique, the cells are damaged by applying force through collisions between the cells and the beads. The efficiency of microalgal cell lysis using this technique largely depends on the bead load and diameter. Typically, zirconia and glass are used as media with high and low viscosity [25].Safi et al. [26] reported that using bead milling for 40 min resulted in a 96% extraction efficiency of total proteins from *Chlorella vulgaris*. On the other hand, Postma et al. [27] reported 32% to 42% efficiency in extracting soluble proteins from the same microalga. Additionally, Günerken et al. [28] studied various cell disruption methods, including bead milling, and achieved protein extraction efficiencies of 60% to 70% in *Chlorella vulgaris*, with efficiency depending on bead load and processing time.

### 4.2. Chemical Extraction

Solvents or detergents are used to dissolve the cell membrane. While effective, this technique carries the risk of certain solvents denaturing proteins or leaving toxic residues that could limit their application in food products [11].

Solvents and chemicals play a crucial role in microalgal protein extraction, each with specific characteristics and uses. For example, organic solvents such as methanol, ethanol, and isopropanol are used to dissolve fats and other lipid components, facilitating protein release. Hexane is often combined with ethanol to efficiently separate lipids and proteins, particularly in *Chlorella vulgaris*. Detergents like SDS (sodium dodecyl sulfate) are highly effective at breaking down resistant cell walls but may be harsh on proteins. In contrast, Triton X-100 is gentler and used when preserving the functionality of sensitive proteins, which is important. Other chemicals like urea, used in high concentrations (6–8 M), help denature proteins, making them more soluble and easier to extract. This method is useful, although it may alter the proteins’ functional properties [15,29,30].

### 4.3. Ultrasound-Assisted Extraction

This technique uses high-frequency waves to generate cavitation, which facilitates cell wall disruption without the need for harsh chemicals [31]. Sankaran et al. [23] used ultrasound as an assisted extraction method, achieving an extraction efficiency of up to 93.33% in *Chlorella vulgaris*. On the other hand, Mittal et al. [32] found that ultrasound alone was not an effective disruption method, but when combined with other techniques, excellent protein extraction yields were achieved from *Gelidium pusillum*. According to their report, maceration combined with ultrasound treatment demonstrated the highest cell wall disruption, resulting in the highest extraction yields (77% and 93%).

### 4.4. Enzyme-Assisted Extraction

Enzymes are used to specifically degrade the cell wall without affecting the proteins. This technique is highly selective and efficient, preserving the functional and bioactive properties of the extracted proteins [26]. However, the cost of enzymes can be a limitation. Recent advancements in enzymatic extraction for *Scenedesmus obliquus* have optimized variables such as enzyme concentration (2–6 µg/mL) and hydrolysis time (2–12 h), achieving extraction efficiencies of 27.87% with a multi-enzyme system (Viscozyme) and 21.42% with cellulase. These yields exceed those obtained through alkaline methods (19.13%), which result in greater protein losses [33]. This is consistent with findings for *Chlorella fusca*, where enzymatic treatments preserved bioactivity more effectively than mechanical disruption [22]. Enzyme-assisted extraction has multiple advantages, such as high selectivity, since enzymes specifically target cell wall components (e.g., cellulose, hemicellulose) This process helps minimize protein denaturation, and the bioactivity, such as the antioxidant, antimicrobial, and anti-inflammatory properties of proteins, remains intact [34,35]. Yield optimization can be achieved through combined strategies, such as enzymatic and mechanical approaches, which enhance yields beyond standalone methods [24,36].

## 5. Impact of Drying, Heating and Enzymatic Treatments

Drying is a crucial step in the extraction of microalgae biomass for protein production. The choice of drying method significantly affects the protein’s quality and functionality, particularly given the sensitivity of certain proteins to temperature. Common drying methods include spray drying, convection drying, and sun drying, each with its own advantages and drawbacks. Therefore, selecting the most suitable method depends on the nature of the microalgae [30,37,38].

In a study by Mendes Costa et al. [24], various treatments applied to *Chlorella vulgaris*, such as drying, extrusion, and enzymatic methods, were evaluated. The results revealed that extrusion led to a threefold increase in peptide concentration due to its mechanical disruption of the algae’s cellular structure. The use of trypsin, in contrast, only enhanced peptide release after combined treatments of lyophilization and pearl milling, indicating a synergistic effect with these pretreatments. Other methods, such as pearl milling and microwave exposure, resulted in the release of protein fractions in the 32–40 kDa range. Pancreatin, however, reduced protein fractions to around 26 kDa after microwave treatment and grinding, and also decreased total protein after sonication. This study demonstrates that the impact on peptides and proteins depends critically on the type of treatment and its sequence within the process.

## 6. Techniques to Increase Protein Content in Microalgae

There are various methods to increase the protein content during the cultivation of microalgae. One such approach is nutrient deprivation or an increase in nutrients during cultivation. Several studies have shown that an increase in nitrate consumption translates to enhanced protein synthesis, reaching approximately 45%, as reported in research conducted with *Chlorella vulgaris* [39].Other methodologies have also been explored, such as optimizing light conditions. Blue light (450–470 nm) has been used to improve photosynthetic efficiency and amino acid synthesis. In *Tetraselmis suecica*, a protein content of 52% was achieved using 200 μmol photons/m^2^/s [40]. Similarly, moderate saline stress can also be used to enhance protein accumulation in microalgae. For instance, a study by Hosseini and Shariati [41] demonstrated that the exposure of *Dunaliella salina* to 25 mM NaCl increased its protein content by 20%.

## 7. Nutritional Properties of Microalgae Proteins

### 7.1. Amino Acid Profile

Microalgae proteins typically exhibit a balanced profile of essential amino acids, making them a promising alternative to animal-based sources [38]. In species such as *Spirulina platensis* and *Chlorella vulgaris*, this profile is notable for a high proportion of essential amino acids (30–50% of the total), with lysine, leucine, valine, and isoleucine being particularly abundant. The former is especially rich in tryptophan, while the latter shows higher levels of methionine. Although the composition varies between species, common patterns emerge: glutamate and aspartate typically make up between 8% and 12% of the total [42]. Among non-essential amino acids, tyrosine, cysteine, glycine, arginine, alanine, proline, serine, and the aforementioned glutamate and aspartate are predominant [43]. This nutritional balance highlights the potential of microalgae as a sustainable resource for supplementing both human and animal diets. On the other hand, Amiri et al. [33] carried out different studies, one of which reported that the proteins of microalgae *Scenedesmus obliquus* exhibit a nutritionally balanced profile of essential amino acids, positioning them as viable alternatives to conventional protein sources. The species is particularly rich in phenylalanine (10.96 mg/g) and leucine (15.82 mg/g), which constitute 30–40% of their total essential amino acids. Notably, leucine levels exceed those found in *Chlorella vulgaris* (15.82 vs. 12 mg/g), aligning with the FAO/WHO reference for adult requirements (59 mg/g). However, methionine was undetected, likely due to degradation during acid hydrolysis, a limitation also observed in other microalgae like *Tetraselmis suecica* [43]. Non-essential amino acids such as aspartic acid (2.76 mg/g) and glutamic acid (10.04 mg/g) collectively represent 8–12% of the total, consistent with patterns seen in *Spirulina platensis* [42].Other non-essential amino acids, including alanine (14.9 mg/g), proline (8.96 mg/g), and arginine (9.1 mg/g), further enhance its nutritional versatility. Despite a chemical index of 0.2–0.4 (lower than animal proteins but superior to cereals), Scenedesmus obliquus demonstrates potential for dietary supplementation, particularly in regions with limited access to high-quality proteins [33,38].

### 7.2. Digestibility and Bioavailability

Digestibility refers to the efficiency with which proteins are broken down into amino acids during digestion. This is a crucial process, as proper protein breakdown leads to better absorption and utilization in the body. Digestibility varies among species and is influenced by several factors, including the composition of the cell wall and the presence of antinutrients. In this regard, the presence of tannins can interfere with protein digestibility by forming complexes that are less soluble and by inhibiting proteases, which reduces digestibility [15]. For example, *Chlorella vulgaris* has a digestibility of around 70–80% due to its rigid cell wall rich in cellulose, which hinders enzymatic breakdown. To overcome this challenge, techniques such as treatments involving mechanical forces have been developed to improve digestibility [44]. Likewise, it has been reported that *Spirulina platensis* shows higher digestibility (80–90%) due to its cellular structure and low cellulose content in the cell wall [45]. In the study conducted by [46], the digestibility of biomolecules, including crude protein, was evaluated across a diverse range of microalgal species. The investigation encompassed *Arthrospira platensis*, *Nannochloropsis sphaeroides*, *Chlorella sorokiniana*, *Chlorella vulgaris*, *Tetraselmis suecica*, *Porphyridium purpureum*, *Phaeodactylum tricornutum*, *Tisochrysis lutea*, and *Nannochloropsis oceanica*. Among the evaluated species, *Nannochloropsis sphaeroides* and *Arthrospira platensis* exhibited the highest crude protein digestibility, achieving approximately one of 80%, followed by *Chlorella vulgaris* (78%), *Chlorella sorokiniana* and *Tetraselmis suecica* (75%), and finally *Phaeodactylum tricornutum* and *Porphyridium purpureum* (71%). These findings underscore the significant interspecies variability in protein bioavailability, with implications for selecting microalgal strains tailored to specific nutritional or functional applications.

### 7.3. Functional Properties of Microalgae Proteins

Proteins extracted from microalgae, despite having limited technological and functional properties due to their high content of insoluble proteins, generally exhibit good emulsifying abilities due to their amphiphilic nature. This is because their structures often contain charged residual amino groups, facilitating favorable interactions with both water and lipid phases, which is essential for forming stable emulsions. Studies on proteins extracted from *Chlorella protothecoides* have shown a poor emulsifying capacity. However, it was observed that their hydrolysis with hydrochloric acid improved solubility and interfacial activity, which could enhance their ability to act as emulsifying and gelling agents [47]}. Another study conducted on soluble proteins isolated from *Chlorella sorokiniana* and *Phaeodactylum tricornutum* revealed considerable interfacial activity in oil–water matrices. Additionally, it was found that 1% Chlorella protein and 3.7% *Phaeodactylum* protein were required to achieve stable emulsions over a period of 7 days [48].

Proteins generally exhibit water-holding properties, which are often essential for their application in food. This characteristic is directly influenced by the hydrophilic amino acid residues present in the protein structure, which enable interactions with water molecules through hydrogen bonds. This water retention property offers several benefits in food applications, including helping maintain softness and moisture in products such as those from the bakery industry [49]. Furthermore, water retention can increase the profitability of processed foods by increasing their weight due to water retention. At the same time, this property prevents syneresis in certain products, such as sausages, which is the process where water separates from the food matrix [50]. According to research by Benelhadj et al. [51], the water retention capacity of protein isolates from *A. plantesis* reached a maximum value of 428.8 g of water/100 g of isolated protein. On the other hand, a study by Zhu et al. [52] found that the water retention capacity (4.06 g of water/g of protein) of *Haematococcus pluvialis* protein was comparable to that of proteins isolated from various legumes, even surpassing that of red lentil protein concentrate, desi chickpea and kabuli chickpea.

Another important functional property of proteins is their ability to form gels. This property is crucial for various food types. It is attributed to the formation of cross-linked polymer networks that can trap water. In this sense, Chronakis [53] evaluated this property, finding that gel formation required the adjustment of the protein isolate to pH 7 and a concentration of 1.5%, using Tris 0.1 M (CaCl_2_ 0.02 M) as a buffer. In another study, Lozober et al. [5], working with the same microalga species, reported that the minimum protein concentration for achieving gelation was 2.5% (*w/v*) at pH 6.5. Meanwhile, Suárez Garcia et al. [54] investigated the gelation behavior of protein isolates from *Tetraselmis suecica* in comparison to whey protein. The process involved heating from 25 °C to 90 °C, resulting in better gelation characteristics compared to whey protein. Additionally, Grossman et al. [55] studied the gelling properties of *Chlorella sorokiniana* protein, finding that gelation began at 61 °C, and applying 80 °C for 10 min was sufficient to achieve gelation using 9.9% (*w*/*v*) protein. On the other hand, Amiri et al. [33] conducted various tests to assess the functional properties of microalgae proteins. *Scenedesmus obliquus* proteins exhibit moderate techno-functional properties, making them suitable for a variety of food matrices. The foaming capacity (FC) was 20%, lower than that of egg white (40%) but higher than that of soy (1%). The fat absorption capacity (FAC) was 124%, surpassing that of egg white (113%) and approaching that of soy (143%). The emulsification capacity (EC) was 100 g oil/100 g sample, outperforming legumes like chickpea [52]. Although the foam stability (25%) lags behind that of egg white (100%), adjustments in ionic strength or pH during extraction could enhance performance in this regard, as demonstrated in *Arthrospira platensis* [51]. These properties support its potential use in baked goods and lipid-rich emulsions.

## 8. Potential Applications in Food Products

Microalgal proteins have a wide range of potential applications in various food types. Due to their exceptional nutritional quality, they could serve as an alternative to meat or other animal and plant-derived proteins. Additionally, these proteins can be incorporated into products such as milk, yogurt, and cheese to fortify them and enhance their stability. They could also be used in the bakery and confectionery industries, as they can improve texture and moisture retention. Furthermore, microalgal proteins may be utilized as dietary supplements. In this regard, microalgal-based protein powders are currently being marketed to help athletes meet their protein requirements more efficiently. Additionally, isolated microalgal proteins may offer bioactive properties, such as antioxidant and antimicrobial activities [15].

The most widely consumed microalga globally is Spirulina, primarily due to its high protein content, as well as its levels of γ-linolenic acid, B vitamins, and phycobiliproteins. The World Health Organization (WHO) has approved this cyanobacterium for consumption, considering it a superfood due to its nutrient density, and it has even been sent to space by NASA. It is estimated that Spirulina contains 180% more calcium than milk, 670% more protein than tofu, 3100% more β-carotene than carrots, and 5100% more iron than spinach [38]. Commercial products like Spiruluka bars and Blue Majik sachets highlight its integration into functional snacks and beverages, providing essential amino acids, antioxidants, and the anti-inflammatory phycocyanin [2,4]. However, products derived from microalgae must comply with various regulations and standards before being designated for human consumption.

International programs exist for the analysis of unconventional foods, but currently, few countries have established legislative standards for the cyanobacterium Spirulina. For other microalgae species, such as *Chlorella* and *Dunaliella*, no official regulations are known. The approval of microalgal biomass for human or animal consumption requires several evaluation steps and specifications, including an assessment of proximate composition, protein quality, essential amino acid content, and toxic and non-toxic substances, as well as sanitary analysis, toxicological evaluation, and safety analysis. Moreover, laws and regulations for food additives, nutraceuticals, and functional foods vary from country to country [43].

In the food industry, carbohydrates derived from microalgae are used as thickeners and gelling agents to improve the texture and quality of food formulations. The physicochemical and biological properties of these carbohydrates depend on their chemical composition, which influences their final application. The type and concentration of carbohydrates vary depending on the species of microalga, with sugars such as arabinose, xylose, mannose, galactose, and glucose, as well as rhamnose, fucose, and uronic acids, being present in their composition. Traditional carbohydrate sources have become more expensive and are declining at an accelerated rate. The use of microalgae holds potential as a sustainable resource, as carbohydrates can make up a significant portion of biomass content, revealing a valuable opportunity for further exploration [56].

An innovative strategy to improve the nutritional value of certain bakery products is the inclusion of microalgal biomass in formulations. In this regard, *C. vulgaris* and *A. platensis* have been used to increase the iron and selenium content in bread, yielding favorable results concerning color and texture. Other species such as *Isochrysis galbana*, *T. suecica*, *Scenedesmus almeriensis*, and *Nannochloropsis gaditana* have also been used in wheat bread formulations, maintaining optimal organoleptic and quality parameters. Furthermore, *H. pluvialis* has been employed as a functional ingredient in whole-wheat cookies, improving glucose release [57]. This is consistent with findings by Kafyra et al. [58], who successfully incorporated lipids and proteins from *C. vulgaris* into a brioche-type bakery formulation, completely replacing butter and eggs from the traditional recipe without affecting sensory or structural attributes. Additionally, the presence of antioxidants and omega-3 and omega-6 fatty acids had favorable effects on the product’s freshness and shelf life.

On the other hand, chlorophyll is used in some industries, being utilized as a color additive (E140) in foods and beverages. It is commercially available in sodium and copper chlorophyllin forms. This conformational change allows the conversion of liposoluble chlorophyll into a water-soluble form, and it is attributed with potential chemopreventive effects, acting as an antimutagenic agent against pro-carcinogenic polycyclic compounds, such as aflatoxin-B1, heterocyclic amines, and polycyclic hydrocarbons [43].

## 9. Bioactive Properties of Microalgae Proteins

### 9.1. Antioxidant Activity

Proteins derived from microalgae have garnered significant interest due to their nutritional and bioactive properties, with their antioxidant capacity being particularly noteworthy. This capacity is crucial for preventing damage caused by reactive oxygen species (ROS), which are linked to cellular aging and the development of chronic degenerative diseases such as cancer and diabetes [59]. Several studies have explored microalgae proteins, highlighting their high natural antioxidant activity. In a study by Bashandy et al. [60], it was revealed that phycocyanin, an active protein taken from *A. platensis*, reduced oxidative stress induced by arsenic at a concentration of 300 mg/kg. It also alleviated testicular damage and sperm abnormalities in male rats. Furthermore, Kang et al. [61] evaluated the antioxidant activity of *Navicula incerta*, finding that its hydrolysates exhibited high antioxidant activity according to DPPH, hydroxyl radical, and superoxide assays, suggesting that proteins from this species could be used as functional food ingredients. Additionally, Alzahrani et al. [36] assessed the antioxidant activity of *Nitzschia laevis* proteins via the ABTS technique, observing that its hydrolysates demonstrated strong antioxidant activity along with the ability to eliminate superoxide anion radicals. Similarly, in a study by Zhang et al. [34], the antioxidant activity of a protein complex from *Chlorella pyrenoidosa* was evaluated. The authors found that this complex could enhance the activity of the antioxidant enzyme superoxide dismutase, indicating promising anti-aging properties. In neurodegenerative disorders, microalgal antioxidants show promise. *Navicula incerta* hydrolysates suppressed hydroxyl and superoxide radicals in vitro, suggesting applicability in Alzheimer’s or Parkinson’s disease management [61]. Additionally, *Spirulina* phycocyanin inhibited neuroinflammation in experimental autoimmune encephalomyelitis models, linking antioxidant activity to neuroprotection [62]. For metabolic diseases, *Nitzschia laevis* peptides reduced oxidative stress markers in hyperglycemic models, highlighting their role in diabetic complication prevention [36]. Microalgae-derived carotenoids, such as *astaxanthin* from *Haematococcus pluvialis*, have been clinically explored for cardiovascular health, reducing LDL oxidation and inflammatory cytokines in atherosclerosis [63]. Furthermore, *Chlorella vulgaris* peptide fractions inhibited ROS-driven DNA damage in human cell lines, underscoring their chemopreventive potential against cancer [64].

### 9.2. Antimicrobial and Antiviral Properties

Antimicrobial and antiviral properties are among the bioactive properties that proteins can exhibit in general. The increasing resistance of microorganisms to antibiotics has highlighted the need to find new sources of active compounds for the treatment of diseases caused by pathogenic microorganisms. In this context, microalgae are of particular interest, as they have developed various antimicrobial tolerance mechanisms, allowing them to withstand exposure to different microorganisms, such as fungi, bacteria, and pathogenic viruses [65,66,67]. Various molecules with antimicrobial potential have been studied, such as eicosapentaenoic acid (EPA) and docosahexaenoic acid (DHA), which have been reported to have strong antibacterial effects against *Streptococcus*, *P. gingivalis*, and *Prevotella intermedia*. These acids have also shown good antifungal activity against *Candida albicans* and other pathogens [68]. However, there is limited information on the antimicrobial effects of proteins extracted from microalgae or their derivatives. In a study by Guzmán et al. [35], peptides derived from *Tetraselmis suecica* were investigated to identify those with antimicrobial activity. Of the three peptides analyzed, AQ-1766 exhibited the highest efficacy against microorganisms. Subsequently, by replacing alanine residues with lysine, six peptide variants were generated with significantly improved antimicrobial activity. The study included tests against several pathogenic bacteria, including *Escherichia coli* ML35, *Salmonella typhimurium*, *Pseudomonas aeruginosa*, *Bacillus cereus*, *Staphylococcus aureus*, *Listeria monocytogenes*, and *Micrococcus luteus*. Additionally, a study by Verdugo [69] demonstrated that protein hydrolysates and peptides derived from *Nannochloropsis* sp. exhibited antimicrobial activity against *Staphylococcus aureus*, *Escherichia coli*, and *Candida albicans*, with inhibition percentages as high as 96.65%.

On the other hand, regarding antiviral activity, studies show that peptides derived from microalgae can effectively inhibit various viruses, including herpes simplex, HIV, and influenza viruses, by altering the lipid membrane or inhibiting critical enzymes for virus replication [70,71]. In this context, a study by Rivera-Serrano et al. [72] evaluated the antiviral effect of hydrolyzed proteins from *Phaeodactylum tricornutum* against the Dengue virus serotype 2. The authors reported that peptide fractions of 10–30 kDa, at concentrations of 150 and 300 μg/mL, significantly reduced the percentage of infected cells. These findings suggest that peptides derived from microalgae display promising antiviral activity against Dengue virus serotype 2. Additionally, Jang and Park [71] reported that a peptide derived from *Spirulina maxima* inhibited HIV-1 infection in a human T cell line (MT4) at a concentration of 0.0691 mM, thus obtaining the median inhibitory concentration (IC_50_). These studies highlight the potential of peptides derived from different microalgae species as antiviral agents against various viruses.

### 9.3. Anti-Inflammatory Effects

Inflammation is a vital biological response of the immune system to external insults such as infections or tissue injuries. However, dysregulated or chronic inflammation can lead to various degenerative diseases, including arthritis, cardiovascular diseases, and certain types of cancer [73]. Microalgae have emerged as a promising source of bioactives due to their metabolic diversity and ability to synthesize a wide range of compounds that are beneficial for health. Among these, proteins and peptides derived from microalgae have shown potential in modulating inflammatory and immune responses. These proteins can interact with various cellular signaling pathways, inhibiting the production of pro-inflammatory mediators such as cytokines and modulating the activity of key immune cells [74,75]. In this regard, Cervantes Llanos et al. [62] orally administered C-phycocyanin, a protein extracted from the microalga *Spirulina* sp., to experimental rats with autoimmune encephalomyelitis, a condition that causes chronic inflammation in the brain. The oral administration of phycocyanin was effective in controlling the condition due to its anti-neuroinflammatory activity. In another study, Zhang et al. [34] used a pigment–protein complex extracted from *Chlorella pyrenoidosa* and evaluated its anti-inflammatory and anti-aging activity. The results were promising, as the pigment–protein complex inhibited the production of inflammatory cytokines such as TNF-α and IL-6, as well as the inflammatory mediator nitric oxide (NO) in RAW 264.7 cells. Additionally, Sheih et al. [64] studied a peptide fraction obtained from the enzymatic hydrolysis of protein waste from *Chlorella vulgaris*, observing that these fractions had the ability to inhibit macrophage production, which is associated with the prevention of inflammation. The various studies analyzed suggest that microalgae not only represent an alternative source for protein extraction but also possess high therapeutic potential in various applications. Therefore, microalgae could play a key role in strategies for disease prevention and treatment, while also contributing to innovation in key sectors such as health, functional food, and environmental sustainability.

## 10. Economic Feasibility

The production of microalgal proteins faces economic challenges related to cultivation, harvesting, and downstream processing. The current cost of dried microalgal biomass (e.g., Spirulina) ranges from $10 to $50 per kg, which is significantly higher than that of soybean ($1–$2 per kg) or whey protein ($5–$10 per kg) [1,7]. The primary cost drivers include a high-energy-consumption process: mechanical disruption methods, such as bead milling and sonication, account for approximately 30% to 40% of total costs. Additionally, scale-up limitations present a challenge, as open-pond systems significantly reduce costs but compromise safety due to the increased risk of contamination in an open environment [3,26,27].

## 11. Environmental Impact

Microalgae production offers sustainability advantages over conventional protein sources due to better land use efficiency. Microalgae produce 10 to 20 times more protein per hectare than soybeans, thus reducing deforestation pressure [56]. Additionally, with respect to their water footprint, microalgae thrive in non-potable or saline water, helping to conserve freshwater resources [1]. Furthermore, regarding carbon sequestration, the photosynthetic fixation of CO_2_ (1.8 kg of CO_2_ per kg of biomass) helps mitigate greenhouse gas emissions [7]. Similarly, with regard to waste valorization, microalgae can utilize industrial CO_2_ flue gasses and agricultural runoff as nutrient sources [11]. Finally, concerning biodegradability, they generate minimal toxic byproducts compared to synthetic fertilizers or chemical solvents [28].

## 12. Research Gaps and Future Perspectives

Although microalgae-derived proteins exhibit great potential, current research is limited by several factors, including methodological heterogeneity. Differences in cultivation conditions (e.g., light intensity, nutrient stress) and the various extraction protocols previously mentioned make direct comparisons of protein yields and functionalities across studies challenging [7,30]. For instance, the protein content of *Chlorella vulgaris* ranges from 40% to 70%, depending on nitrogen availability, but standardized cultivation procedures are lacking [11,39]. Further complicating progress is the limited understanding of how microalgal protein structures such as phycobiliproteins and glycoproteins interact with human metabolic pathways to exert bioactivity. Additionally, there remains a scarcity of data on long-term safety concerns, including chronic toxicity, allergenicity, or interactions with pharmaceuticals, which are critical for clinical translation [43,76]. Finally, while integrated biorefineries could enhance economic feasibility by co-producing proteins, biofuels, and high-value pigments (e.g., *astaxanthin*), few studies have explored such circular systems to offset production costs [9].

## 13. Conclusions

In the face of global challenges related to food security and sustainability, microalgae emerge as a revolutionary option. Their ability to grow in unconventional environments, with photosynthetic efficiency up to ten times higher than that of terrestrial plants, makes them ideal candidates for producing high-quality proteins. Species like *Arthrospira platensis* not only match the protein content of meat but also that of surpass some plant sources like soy, while offering essential amino acids for human nutrition. However, their extraction or recovery remains one of the main challenges, prompting the development of combined techniques (mechanical, enzymatic, or physical) that achieve yields greater than 90% without compromising functionality. Nutritionally, their digestibility varies by species, with most showing relatively high levels of digestibility. These characteristics, alongside emulsifying and water retention properties, allow their integration into everyday foods, from bread to dairy alternatives. Beyond nutrition, some peptides derived from microalgae demonstrate unique bioactive properties, such as combating bacterial pathogens or even inhibiting viruses like Dengue, opening doors for pharmaceutical applications. However, large-scale utilization faces significant hurdles, including ambiguous regulatory frameworks, high production costs, and the need to educate consumers about their benefits.

## Data Availability

No new data were created or analyzed in this study. Data sharing is not applicable to this article.
